# Value of ultrasound in detecting subclinical synovitis in polyarticular juvenile idiophatic arthritis (PJIA) in clinical remission (CR)

**DOI:** 10.1186/1546-0096-12-S1-P165

**Published:** 2014-09-17

**Authors:** Silvia Meiorin, Maria Laura Barzola, Graciela Espada, Lidia Blumenthal

**Affiliations:** 1 Hospital De Niños Ricardo Gutierrez, Buenos Aires, Argentina

## Introduction

Ultrasonography (US) is a useful tool to determine synovial inflammatory involvement, anatomical damage, and “subclinical synovitis” in adult and juvenile pts with RA.

## Objectives

To describe US findings in pJIA pts in CR.

## Methods

Prospective study.

## Inclusion criteria

pJIA patients (ILAR'01) with inactive disease (according to Wallace criteria). All patients underwent an ultrasound assessment within the week when clinical examination was done. The U.S. examiner was blinded to clinical findings and the US were carried out on previously affected joints. A Toshiba equipment, model Nemio, transducer 6-12 Hz was used. The following findings were considered pathological in U.S: synovial hypertrophy, effusion and tenosynovitis (by gray scale) and positive Power Doppler (PD). Subclinical synovitis was defined as synovial hypertrophy and positive PD, in absence of clinical arthritis. Demographic, clinical- functional, laboratory and therapeuthical variables were analyzed.

## Results

A total of 19 patients were included, 15 females (79 %), median age 12.9 years (IQR 11.4-6.2), median disease duration 6.04 years (IQR 3.5-8.5) and median remission period 1.53 years (IQR 1.18-2.32). Sixteen out of 19 patients (84%) were in CRM (17 MTX, 6 anti-TNF), and 3 in CR. The CHAQ score mean value was 0.02 (SD ± 0.06). Two hundred joints were systematically evaluated (clinical + US): structural alterations were found in 11/19 (58%) pts and in 14/ 200 (7%) joints (see table 1).

**Table 1:** 

Joints	Total assessd n=200	US anormalities n=14 (7%)	Findings (gray escale)	Power Doppler
Wrists	30	2(6)	Tenosynovitis2	-

Pifs	70	1(1,4)	Effusion1	-

Mcfs	40	3(7,5)	Effusion3	-

Knees	24	3(12,5)	Sinovial hyperplasia3 Effusion1 Cyst1	-

Ankles	36	5(14)	Sinovial hyperplasia3 Tenosynovitis2	-

## Conclusion

In our series, subclinical synovitis was not detected by U.S. The prevalence of abnormalities by grayscale was 58% pts (11/19), these findings suggest the possibility of persistent inflammation (in US exam) in spite of the absence of clinical finding related with “active disease”.

## Disclosure of interest

None declared.

